# Frontiers in the Risk Stratification and Prevention of Sudden Cardiac Arrest and Death in Athletes

**DOI:** 10.3390/jcm15207920

**Published:** 2026-10-14

**Authors:** Jacob Cushing, Luke Hamar, John Fritzlen

**Affiliations:** 1 Department of Cardiovascular Medicine, The University of Kansas Medical Center, Kansas City, KS 66160, USA; jcushing2@kumc.edu; 2 Department of Family Medicine, The University of Kansas Medical Center, Kansas City, KS 66160, USA; lhamar@kumc.edu

**Keywords:** athletes, sports cardiology, screening, sudden cardiac death

## Abstract

**Background**: Sudden cardiac arrest (SCA) and sudden cardiac death (SCD) remain the leading causes of exercise-related mortality in athletes. Research in recent years has produced substantial advances in the understanding of athlete-specific risk, the etiologic spectrum of SCA/SCD, and the tools available for screening and risk stratification. **Methods**: We performed a narrative review of the literature identified through PubMed/MEDLINE searches of related terms, supplemented by targeted searches and reference checking. **Summary of the Review**: In this narrative review, we synthesize updated evidence on the epidemiology, etiologies, screening, and emerging technologies relevant to SCA/SCD in athletes. **Conclusions**: Contemporary evaluation of SCA/SCD risk in athletes relies on multimodal, individualized assessment with longitudinal surveillance and shared decision-making. Emerging computational tools may further refine risk prediction, but current evidence remains limited.

## 1. Introduction

There have been many advancements in the study of sudden cardiac arrest (SCA) and sudden cardiac death (SCD) in athletes in the past several years. As we learn more about the physiologic changes that occur in the athlete heart, we have become better at identifying maladaptive processes that place the athlete at risk. In this review, we discuss recent updates to the care of athletes with or at risk of SCA/SCD. We explore contemporary incidence estimates of SCA/SCD in athletes compared to the general population, disease-specific advancements, and guidance on return to play. We address the initial response to an arrest: recognition, immediate CPR, AED access, and comprehensive survivor evaluation. We then review updates in screening and indications for ICDs and recent technologic advancements. We conclude with a brief review of the evolving role of artificial intelligence (AI) and its potential impact in future research.

## 2. Materials and Methods

This work is a narrative review of the contemporary literature on SCA and SCD in athletes. The authors’ last PubMed/MEDLINE (U.S. National Library of Medicine, Bethesda, MD, USA) literature search was performed on 27 September 2026, with an emphasis on articles published from 2025 onward and the inclusion of earlier historical and foundational studies as needed. Included articles were available in English or in English translation. The original search combined sudden cardiac arrest, sudden cardiac death, athlete, and artificial intelligence with terms for individual diseases, including myocarditis and dilated cardiomyopathy. Targeted revision searches added competitive sport, endurance exercise, screening, electrocardiography, return to play, coronary anomaly, pre-excitation, channelopathy, resuscitation, emergency action plans, AEDs, survivor evaluation, genetic testing, and computational heart models. References and publication details were checked against original articles, PubMed records, and society documents through 27 September 2026.

Selection was purposive rather than exhaustive. Priority was given to peer-reviewed original research, meta-analyses, registry and autopsy series, and current society guidelines, with an emphasis on recent publications in the past 1–2 years. Reference lists of retrieved articles were hand-searched for additional relevant sources. Because this is a narrative rather than a systematic review, no formal protocol registration, predefined inclusion/exclusion criteria, quantitative synthesis, or risk-of-bias assessment were undertaken. Articles were selected by the authors for clinical relevance and topical currency. Reference lists of papers found using the above methods were used to locate additional relevant work as needed—primarily for papers published before 2025. Finally, following peer review, several more references were added from reviewer suggestion. No human or animal subjects were involved, and no generative artificial intelligence was used to generate the scientific content of this manuscript.

No proprietary software, instruments, laboratory reagents, or devices were used in the conduct of this narrative review beyond the literature databases identified above.

## 3. Definitions and Scope

The review includes adolescent and adult competitive athletes and people undertaking sustained recreational or endurance training. Several definitions are key to understanding this document. A competitive athlete trains for organized competition; recreational participants are considered separately because training exposure, screening requirements, and access to medical care differ. Masters athletes are generally aged 35 years or older, although the threshold and training definition used by each source are retained when interpreting that study. Pediatric referral cohorts, military populations, and community samples are not treated as interchangeable with athlete cohorts [1,2,3]. Sudden cardiac arrest (SCA) refers to the sudden cessation of normal cardiac activity with hemodynamic collapse, while sudden cardiac death (SCD) is defined as the sudden natural death by a cardiac cause within 1 h of the onset of symptoms or within 24 h from the time last seen alive for unwitnessed events [4]. SCA denotes the arrest event itself, which may be survived with timely recognition and defibrillation, whereas SCD denotes the fatal outcome. We use the term autopsy-positive sudden death to refer to a death accompanied by a structural cardiac cause, while autopsy-negative sudden unexplained death (AN-SUD) is used when a comprehensive autopsy does not reveal a structural cardiac cause. We will use “concealed” or “hidden” substrate to refer to an underlying arrhythmogenic cardiac abnormality that is clinically unapparent on routine evaluation but predisposes one to sudden cardiac arrest. Examples of the hidden substrate include channelopathies, microscopic myocardial fibrosis, and myocarditis [5,6].

The scope of this document includes a review of the current literature and a look into several key topics with recent developments. We discuss risk factors, screening considerations, autopsy findings, and more amongst various population groups. Subpopulation groups include pediatric patients, female athletes, and endurance and masters athletes. We also review updates to specific conditions, including new work on arrhythmogenic cardiomyopathy (ACM), hypertrophic cardiomyopathy (HCM), and several channelopathies. Additionally, the increasing volume of AN-SUD yields a look into the concealed (or hidden) substrate. We discuss updated screening guidelines with ECG and targeted imaging in connection with channelopathies, coronary anomalies, and other barriers to detecting underlying cardiac risk pathologies. Finally, we look to the future by discussing the incorporation of deep learning and artificial intelligence (AI) with future risk predictions, analysis of current data, and integration with different forms of media. By this discussion, we aim to lay the groundwork for continuing improvements in the understanding and management of sudden cardiac arrest and death in athletes.

## 4. Contemporary Epidemiology: Incidence Depends on the Denominator

The incidence of sudden cardiac death varies amongst different groups and classifications of athletes. The central reason for this variation is two-fold: certain demographics carry unique risk factors for cardiac arrhythmias, and people compete at significantly different levels and types of activity. Age, sex, and ethnicity all contribute to underlying pathology. Exercise intensity and duration are associated with cardiac remodeling and coronary artery calcification, although direct evidence that these changes increase SCD incidence is limited [7,8,9]. Interestingly, as survival has increased from sudden cardiac arrest with the higher rate of defibrillator use, the literature increasingly reports SCA and SCD differently regarding incidence among different groups [10,11].

Incidence varies with age and sex. SCD in younger athletes (younger than 25) is more likely to originate from congenital or inherited pathologies than SCD in older athletes, which is more likely to involve coronary artery remodeling with acute injury [1,10,12]. Incidence is higher in males and older athletes: in cohorts with a defined population at risk, males have a 4-to-6-fold greater incidence of SCA/SCD than females, whereas the observation that males account for approximately 80–90% of cases describes case composition and does not by itself establish sex-specific incidence [13,14,15]. In a recent case series, females accounted for approximately 16% of SCA/SCD cases and males for 84%; without participation denominators, these proportions describe case mix rather than relative risk [13]. For community context, Couper et al. [16] reported a median SCD incidence of 1.7 per 100,000 person-years (IQR 1.3–2.6) across general-population studies of individuals aged 12–39 years, with higher incidence in males and older individuals; this is not an athlete-specific estimate. When evaluating the distribution of causes among school-age athletes in the United States, middle-school athletes are more likely to suffer from SCA/SCD secondary to coronary anomalies, while high-schoolers are more likely to suffer cardiac death from hypertrophic cardiomyopathy [17].

Observed incidence of SCA/SCD differs by sport and competitive level. In the 2002–2022 National Collegiate Athletic Association (NCAA) study, 143 SCDs yielded an overall incidence of 1 per 63,682 athlete-years, and the highest incidence was in Division I male basketball players (1 per 8188 athlete-years); these rates include deaths at any time, not only during exercise, and should not be generalized to other levels of basketball participation [17,18]. Even so, the overall incidence of SCD remains low even in high-risk sport participation. Direct outcome evidence that greater exercise exposure increases SCD incidence is lacking. Masters endurance athletes demonstrate a higher prevalence of coronary artery calcification and atherosclerotic plaque burden than age-matched active controls, despite otherwise favorable cardiovascular risk profiles, although these imaging findings are surrogate markers and do not by themselves establish an increased risk of SCD [8,9,19]. Kim et al. ([20,21]) used the RACER prospective observation case series of US marathon runners to determine the incidence of race-associated SCA and SCD among marathon and half-marathon finishers in the RACER 1 (2000–2009) and RACER 2 (2010–2023) studies. In RACER 1, HCM was the most common cause in cases with a definitive etiology in this population, and in RACER 2 it was CAD [20,21]. In RACER 2, the incidence of race-associated SCA was similar to that in RACER 1 (0.60 vs. 0.54 per 100,000 participants), whereas the incidence of fatal SCD declined (0.20 vs. 0.39 per 100,000 participants), reflecting improved survival rather than a rising incidence of arrest [21]. Acute exertional triggers superimposed on chronic remodeling are a proposed mechanism for race-associated events in this population [7,10].

Despite differences in SCD incidence among populations, the overall incidence of sudden cardiac death remains low across reported populations. Based on a recent meta-analysis of SCD incidence across all levels of competition, the pooled incidence of fatal SCD was estimated at 0.98 per 100,000 athlete-years (95% CI, 0.62–1.53), and three studies of competitive athletes aged 14–25 years yielded 1.91 per 100,000 athlete-years (95% CI, 0.71–5.14); these are heterogeneous fatal-event estimates rather than universal rates [15]. Understanding contemporary etiology with stratification of incidence based on demographics and exercise exposure helps to individualize screening and precautionary plans.

Recent autopsy analysis of the Postmortem Systemic Investigation of Sudden Cardiac Death (POST SCD) by Nakasuka et al. ([22]) and Salazar et al. ([23]) provides updated community autopsy data on the etiologies of SCD in a general adult population. POST SCD is a San Francisco county-wide adult cohort without an athlete arm, so it cannot support direct athlete-versus-community risk comparisons and is retained here as background context only [22,23]. In their analysis of 877 autopsies with presumed SCD and health records for review, Salazar et al. found that 513 (58%) were from arrhythmic death. They found that only 166 (32%) had prior diagnosed risk factors (defined as low EF, heart failure, myocardial infarction [MI], syncope) and that 159 (31%) died from occult MI or dilated cardiomyopathy (DCM). Only 3 had structurally normal hearts [23]. Nakasuka et al. used the same POST SCD population to study 943 patients with county-wide presumed SCD cases and found that of those who had autopsy-confirmed SCD (583), 41% (237) experienced SCD due to MI [22]. Among young athletes, contemporary autopsy series identify AN-SUD (~20%) as the most common postmortem classification, followed by HCM (~12%) and idiopathic left ventricular hypertrophy (LVH), fibrosis, or cardiomyopathy not otherwise specified (~11%) [10,18]. With increasing age, however, the athlete phenotype converges with that of the general population, as premature coronary artery disease (CAD), prior MI, and ischemic scar become increasingly prevalent [1,24]. Thus, young-athlete SCD is disproportionately classified as AN-SUD, a postmortem classification rather than a proven electrical etiology, whereas community SCD in older adults is usually associated with occult or recognized structural and ischemic pathology.

## 5. Female Athletes and Sex Differences

Studies have consistently shown lower rates of SCD in female athletes than in males [13,14,25]. The percentages reported below describe the distribution of etiologies among affected athletes and should not be read as sex-specific incidence, which requires participation-based denominators. While the specific etiology of the differences between the incidence in SCA/SCD is unknown, several patterns have emerged. Using two different databases over a 21-year period, Petek et al. ([13]) recently analyzed cases of SCD and SCA in athletes. They found that women who experienced SCA/SCD were significantly more likely to have coronary artery abnormalities (21.3% versus 11.1%, *p* = 0.03), whereas the lower rate of myocardial disease in women, after excluding idiopathic left ventricular hypertrophy, did not reach statistical significance (19% versus 30%, *p* = 0.056). They also found that female cases of SCA/SCD were more often associated with Long QT (12.0% females, 4.0% males) and AN-SUD (17.3% females, 10.8% males) [13]. This is similar to previous studies, concluding that there are lower rates of myocardial disease in women [14,26,27]. From a UK autopsy referral registry of 7675 sudden deaths, Finocchiaro et al. ([27]) studied 756 adolescent decedents aged 10–19 years, of whom 128 (17%) were competitive athletes, and found that myocardial disease was a less common cause of SCD in women; they found, though, a more common finding of normal autopsy indicative of sudden arrhythmic death syndrome (70% of deaths in women, compared to 62% of males) [27].

Differences in ECGs have been noted between men and women athletes. In a review of a registry including 4423 athletes, Orchard et al. ([28]) found a higher rate of T-wave inversions in women compared to men (1.5% versus 0.3%), which led to a higher rate of abnormal screening ECGs overall. Follow-up imaging showed no significant differences [28]. This study, among others, has not found an association between these changes and an increase in SCA or SCD. These findings were replicated in a meta-analysis by Davis et al. ([25]), who also reported a 16 milliseconds (ms) longer QT in women versus men. More research is needed to determine clinical outcomes from these ECG differences.

## 6. Inherited and Congenital Heart Disease

As mentioned above, athletes younger than 25 are at an increased risk of SCD due to arrhythmogenic conditions, simple congenital abnormalities, and complex congenital abnormalities. Screening the pediatric population for risks of sudden cardiac arrest and sudden cardiac death relies on family history and ECG if needed. A family history that includes SCA or unexplained sudden death in a relative younger than 40 years, a diagnosed cardiomyopathy or channelopathy, unexplained exertional syncope, drowning, and early-onset conduction disease or pacemaker requirement should prompt additional evaluation and targeted screening. ECG screening can be challenging due to physiologic changes in heart rate, QRS axis, ventricular predominance, intraventricular conduction, and repolarization morphology. ECG characteristics evolve throughout childhood and adolescence, requiring age-appropriate criteria and ranges rather than a single threshold [29,30]. Genetic testing may be considered depending on family history. It is most informative when targeted and should begin with an affected relative, or post-mortem tissue from a decedent, who has a clinically established or strongly suspected phenotype. Broad panel testing in an athlete without a phenotype or an identified familial variant has low diagnostic yield and risks misinterpretation of variants of uncertain significance [4,5]. A positive genotype can aid in the understanding of a particular phenotype and can provide further risk stratification.

When approaching patients with congenital disease, considerations for underlying anatomy as well as exercise testing help to inform individual exercise capacity [31]. Stratifying patients into higher-risk and lower-risk groups provides a frame of reference for participation recommendations. Shared decision-making should be utilized when creating individual exercise plans regardless of the underlying congenital condition.

Both the ACC/AHA and the EAPC/ESC/AEPC have provided guidelines for participation recommendations for those with inherited and/or congenital heart disease [32]. Overall, recommendations for sports participation in athletes with inherited or congenital disease are individualized based on underlying pathology and the degree of the defect. Recommendations are reflected in the 2025 ACC/AHA Clinical Considerations for Competitive Sports, which bases competitive participation on residual anatomy and physiology, ventricular function, arrhythmias, oxygenation, functional capacity, and the demands of training and competition, with shared decision-making and continued surveillance [33]. While advice for recreational activity is discussed based on exercise testing using shared decision-making discussions, these guidelines from the ACC/AHA help when considering participation in competitive sports.

Simple congenital malformations, such as atrial septal defect (ASD) and ventricular septal defect (VSD), typically cause minimal to mild symptoms depending on the hemodynamic significance of the shunt. Participation recommendations for simple shunting lesions largely depend on the hemodynamic significance of the shunt. Athletes with ASD and VSD should be assessed for pulmonary arterial hypertension (PAH) to guide decisions for medical therapy and exercise tolerance to improve morbidity and mortality [34]. Coarctation is another pathology that requires further evaluation using the resting systolic blood pressure gradient between the upper and lower extremities, exercise testing for the peak systolic blood pressure response (exercise-induced hypertension), and imaging assessment of the ascending aorta size as well as the coarctation/repair site for aneurysm or recoarctation and any associated aortic valve disease [34].

Complex congenital heart conditions include tetralogy of Fallot, univentricular physiology (Fontan circulation), and transposition of the great arteries with atrial baffle repair [35]. Complex lesions involve more severe disease, leading to more pronounced symptoms and potential deoxygenation. As SCD risk is highest in this complex group, it is particularly important to individualize recommendations based on underlying conditions, degree of residual defect, and demand of proposed sport [36]. Athletes with repaired tetralogy of Fallot may experience pulmonary valve dysfunction with residual RV hypertrophy, atrial arrhythmias, or ventricular dysfunction. If considerable defects are present, lower static and lower dynamic exercises are recommended. Competitive sport participation in these athletes should take into consideration these residual defects with longitudinal exercise testing and ambulatory rhythm monitoring. Those with post-Fontan palliation are at an even higher risk of SCA/SCD due to the complex anatomy and low exercise tolerance in this population. Assessment of these patients should include imaging to visualize anatomy and exercise testing to assess hemodynamics and oxygen capacity. Due to the complex anatomy and residual effects of exercise tolerance, these patients are commonly limited to low static, low-dynamic sports. Finally, athletes with repaired transposition of the great arteries have stricter recommendations ranging from limited activity to mild to moderate strength and endurance sports, due to the danger of ventricular dysfunction and arrhythmia development [2,35,37].

## 7. Anomalous Coronary Arteries

Anomalous aortic origin of a coronary artery (AAOCA) is an important substrate in young-athlete arrest series, although its proportion varies with the cohort and endpoint [10,17,18]. Recent advancements in imaging and surgical techniques, such as unroofing, have helped in management of these cases [38]. The combination of transthoracic echocardiography (TTE) to assess coronary origins along with coronary computed tomography angiography (CCTA) to define uncertain or high-risk anatomy has allowed for the detection and risk evaluation of more cases [39].

In adults in the general population with suspected AAOCA, the 2025 ACC/AHA congenital heart disease guideline recommends CCTA, or magnetic resonance angiography when computed tomography (CT) is contraindicated, to confirm the anatomic diagnosis and guide management, followed by risk stratification with evaluation for physiological and/or ischemic changes when the anomalous artery arises from the opposite sinus [34]. Proposed high-risk phenotypes include an intramural course, high take-off, or slit-like proximal orifice in interarterial anomalous aortic origin of the left coronary artery (AAOLCA) and a longer intramural segment in interarterial anomalous aortic origin of the right coronary artery (AAORCA); however, many patients with these features never experience SCA or SCD, and research is ongoing to determine which phenotypes truly correlate with clinical outcomes [34,37]. Other AAOLCA subtypes, such as an intraseptal course or origin from the noncoronary sinus, are generally well tolerated and usually compatible with continued competition, although risk stratification is still warranted because they can rarely present with SCA or myocardial ischemia [37].

Surgery is recommended for patients with symptoms or diagnostic evidence of myocardial ischemia attributable to the AAOCA and is reasonable in asymptomatic patients with an anomalous left coronary artery and high-risk anatomy. In asymptomatic patients without evidence of ischemia or compromised coronary perfusion, the benefit of surgery is not well established, and either continued observation or surgery may be reasonable [34]. After repair, return requires sternal healing and assessment showing no inducible ischemia or complex ventricular arrhythmia [34,37]. New techniques are being developed to help detect and classify high-risk features beyond traditional methods with the use of AI [40], but more research is needed to determine if this can be applied to athletes and affects return to play.

In cases of anomalous origin of a coronary artery from the pulmonary artery, patients typically present with heart failure in the first year of life and require surgical repair. Presentation in later childhood or adulthood is rare. After repair, competitive sports participation is reasonable once sternal healing is complete, provided the ischemic evaluation and left ventricular function are normal and there is no inducible ventricular arrhythmia [37].

## 8. Concealed Substrate and the Impact of Myocardial Fibrosis

AN-SUD remains one of the most common postmortem classifications in athletes, contributing 19–20% of cases between the ages of 18 to 39 years old [10,18]. In these structurally normal hearts, postmortem genetic testing has given us insight into possible mechanisms by identifying cardiomyopathy and arrhythmogenic associated genes. Analysis from UK registry data yielded pathogenic and likely pathogenic variants in 21% of athletes undergoing testing and clinically actionable variants in 15% of athletes with structurally normal hearts [41].

Idiopathic left ventricular fibrosis was also found in athletes and is an important area of ongoing research. Recently, Javed et al. ([7]) prospectively studied 106 masters male athletes over a median of 796 days. These participants underwent exercise monitoring, cardiac MRI (CMR), and received an implantable loop recorder. Of the 106 participants, 25 (23.5%) experienced at least one ventricular arrhythmic episode–22 (20.8%) developed nonsustained ventricular tachycardia (NSVT) and 3 (2.8%) developed ventricular tachycardia (VT). Those athletes who experienced ventricular arrhythmia had a significantly greater prevalence of myocardial fibrosis than athletes without ventricular arrhythmia. Moreover, all athletes with sustained VT had myocardial fibrosis on CMR. While this study is limited to a specific and older athlete population, and not representative of a younger and more diverse athlete cohort, it suggests that myocardial fibrosis may be an arrhythmogenic substrate [7,42].

In a similar cohort of primarily male masters athletes, Zorzi et al. ([6]) performed CMRs on 245 asymptomatic athletes referred to Sports Cardiology who had confirmed ventricular arrhythmias (often premature ventricular contractions [PVCs]) on pre-participation screening. Of those, 110 had LV late gadolinium enhancement (LGE), and among those, 40 had nonischemic left ventricular scar in 2 or more segments and were available for genetic testing with follow-up. They found that, on average, these athletes were male, with negative genetic testing, right bundle branch block (RBBB) morphology arrhythmias, mild resting ECG abnormalities, and normal echo [6].

It is possible that even more cases are affected by concealed substrate than detected by ECG or CMR alone in structurally normal hearts. Haissaguerre et al. ([43]) performed electrophysiological (EP) mapping on 104 patients over the age of 15 who experienced unexplained/idiopathic ventricular fibrillation (IVF). In their study, IVF was diagnosed after negative coronary examinations, genetic testing, and cardiac imaging. In those cases, 19 (18.2%) of IVF occurred during sport, 13 of whom had negative toxicology reports. Despite normal prior work-up, EP mapping of those with sport-related ventricular fibrillation (VF) showed that 73.7% (14) had microstructural cardiomyopathies, compared to 31.8% (27) of those with IVF outside of sports. They additionally noted that provocation using isoproterenol was more effective at inducing significant arrhythmias than the standard exercise test. They concluded that their findings suggest that pre-existing permanent substrate, rather than an elusive factor, contributes to sports-related VF [43]. These were selected arrest survivors undergoing invasive mapping, not a screening cohort. More work is needed to clarify and characterize these microstructural changes, and how they differ from the physiologic changes seen in athlete hearts [44].

Fibrosis assessment should integrate symptoms, family history, LGE distribution and extent, ventricular function, sport-specific exercise testing, and ambulatory ECG monitoring. Limited data are available on return to play in athletes with myocardial fibrosis on CMR. The final decision is based on shared decision-making [2,37].

## 9. Coronary Artery Disease

While exercise is thought to be beneficial for cardiometabolic profile and mortality, it is possible that high-volume exercisers may have reduced benefit in CAD reduction or even increased burden. To specifically study the rate of CAD in athletes in 2008, Mohlenkamp et al. ([45]) studied 108 healthy male marathon runners 50 years or older. They found similar rates of coronary artery calcium (CAC) scores 100 or higher in 36% of runners, similar to age-matched controls. They further noticed that higher CAC scores may be associated with a higher rate of events compared to controls [45]. Similarly, Aengevaeren et al. ([19]), in a cross-sectional study with a retrospectively reported lifetime exercise volume of 284 men engaged in competitive or recreational leisure sports found increasing CAC scores, plaque burden on CTA, and less CAC = 0 as athletes approached > 2000 metabolic equivalent of task (MET)-min/week [19]. Merghani et al. ([46]) even found higher median CAC scores in those with coronary calcium, CAC 300 or higher, and higher plaque quantity of masters athletes compared to sedentary controls. One hypothesis for these findings is that higher hemodynamic stress can lead to increased coronary flow turbulence, endothelial dysfunction, and inflammation [3,46,47,48].

Contrary to these findings, in a cross-sectional study of 23,383 men, Pavlovic et al. ([49]) found decreased CAC scores and decreased rates of CAC 100 or higher with higher intensity regimens. They did note, however, that total exercise volume was associated with an increased number of coronary calcified lesions [49]. Other studies have also suggested minimal/no adverse effects of exercise intensity or duration on coronary plaque burden [48]. A recent 2026 systematic review of CAD in Masters athletes by Gill et al. ([3]) sought to help clarify these discrepancies. They examined 17 studies covering CAD in 2749 masters athletes. They concluded that masters athletes are not immune to the effects of CV risk factors, and the presence of obstructive disease is more likely to have a clinical impact, as fixed coronary stenosis could limit myocardial perfusion during exercise [3]. Ultimately, more work is needed to clarify the relationship between exercise intensity and clinically meaningful CAD outcomes [49]. Most of the trials above have reviewed middle-aged, white male athletes with endurance/aerobic exercises. Furthermore, data remains limited relating CAC and plaque burdens to CAD outcomes compared to sedentary controls [3,49].

## 10. Hypertrophic Cardiomyopathy

Hypertrophic cardiomyopathy is a common inherited disease encountered in clinical practice. Recent evidence suggests that it contributes 12–16% of SCA/SCD in athletes [10,18]. Previously, patients were discouraged from exercising due to risk for SCA. However, recent studies have demonstrated variability in risk, and some evidence that sedentary lifestyle increases long-term risk with worsening cardiometabolic health [50,51]. Those who are genotype-positive yet phenotype-negative may have particularly lower risk than previously thought [51,52].

The primary mechanisms of SCA in HCM are ventricular fibrillation and ventricular tachycardia [53]. Thorough risk assessment and counseling should be performed by an expert in hypertrophic cardiomyopathy with efforts to mitigate risk. When managed appropriately, athletes may have the opportunity to continue to compete [37]. A small study examining return-to-play outcomes in elite-level athletes with genetic conditions, including HCM, did not find a greater number of breakthrough cardiac events with exercise [54]. These are small, retrospective, largely self-referred series without comparator groups; they show that events are uncommon in selected athletes but cannot exclude a clinically important increase in risk. The therapeutics of obstructive HCM have advanced, with widespread adoption of cardiac myosin inhibitors to reduce left ventricular outflow tract (LVOT) obstruction, improve functional capacity, and improve quality of life. Though the European Society of Cardiology (ESC) risk calculator utilizes LVOT obstruction as part of risk analysis, cardiac myosin inhibitors have not been studied to reduce the risk of SCA [55]. Cardiac myosin inhibitors have been successfully used in at least one elite athlete with HCM and obstruction [56], paving the way for future investigation into their potential to reduce the risk of SCA in HCM patients with obstruction. Currently, the AHA and ACC recommend mild to moderate intensity exercise in patients with HCM and allow participants to consider high-intensity physical activity in shared decision-making [37,57].

## 11. Dilated Cardiomyopathy

DCM accounts for approximately 2% of SCA/SCD in athletes [10,18]. DCM can incorporate a large spectrum of genotypes and phenotypes which makes assessing the risk of SCA in DCM challenging. Traditionally, low-risk athletes were thought to be those with LVEF > 45%, absence of arrhythmias on monitoring, absence of LGE on CMR, preserved contractile reserve and absence of high-risk genotypes [37,58,59]. One of the most important genotypes to identify is lamin A/C, as competitive athletes and recreational athletes have demonstrated progressive LV systolic dysfunction compared with more sedentary individuals [60]. Those individuals with a filamin C (FLNC) mutation are also considered to have a higher-risk genotype. It may be reasonable, however, that athletes who are genotype-positive, phenotype-negative consider competitive sports participation after discussing some of the unknown risk of progression. Lower-risk genotypes include titin (TTN). Should an athlete have a decline in LV systolic function < 40% or symptoms, repeat risk assessment needs to be performed with updated imaging and rhythm monitoring to provide further counseling on increased risk [37].

## 12. Arrhythmogenic Cardiomyopathy

Arrhythmogenic cardiomyopathy (ACM) is often associated with ventricular arrhythmias out of proportion to the level of cardiac dysfunction of the right, left, or both ventricles. Historically, data have been limited to inform recommendations and risk prevention of SCA in patients with genotype-positive, phenotype-negative disease [37,61]. In a recent longitudinal cohort study, Pergola et al. ([61]) performed genetic testing on 533 individuals between 2005 and 2024 as part of the TRIAD registry. They found that male sex (HR 1.7), desmoplakin (DSP) mutations (non-missense HR 2.3, missense in hotspot domains HR 2.7), plakophilin-2 (PKP2) missense mutations (HR 3.6) or double-hit mutations (HR 3.4) each had increased risk of life-threatening arrhythmic events. Furthermore, they found that DSP mutation carriers were more likely to experience SCD as the first manifestation of disease [61]. These authors argued for the increased use of a genotype-based approach to risk stratify SCD, as opposed to phenotype-based inclusion. While it is currently reasonable for athletes who are genotype-positive, phenotype-negative to participate in sports, close monitoring and thorough risk discussions of developing ACM with ventricular arrhythmias should be performed.

As it pertains to athletes with PKP2 mutation, intensity (>6 METs) rather than duration has been associated with ventricular arrhythmias [62], so athletes should be advised of the potential harms of high-volume, intense exercise even in the absence of phenotypic expression [37,57,63].

There is limited data on outcomes in return to play for athletes with desmosomal mutations. However, a recent publication examining breakthrough cardiac events for 138 genotype-positive athletes with desmosomal arrhythmogenic cardiomyopathy found an overall event rate of 25% in this cohort. This group included phenotype-positive and phenotype-negative individuals. At follow-up, approximately 15% of phenotype-negative athletes had converted to phenotype-positive. The event rates were higher among those with phenotypic expression compared to phenotype-negative athletes [64]. This was a small, retrospective, single-center study without comparator groups. And yet, the event rate was significant in these athletes and the results could help guide risk-benefit conversations with patients. Further studies are needed to refine risk and guide sports participation.

## 13. Wolff-Parkinson-White

Wolff-Parkinson-White (WPW) pattern is the finding of ventricular pre-excitation on ECG, whereas the syndrome is both ventricular pre-excitation and symptomatic arrhythmias. Although rare, the first manifestation of WPW syndrome can be SCD and has mainly been described in children [65]. Recently, WPW syndrome contributed to 1–3% of SCA/SCD events in athletes ages 18–26 [10,18,66], and individuals with WPW in the general population carry a 3–4% lifetime risk of SCD [67]. Jemtrén et al. ([67]) evaluated the use of exercise stress testing to risk stratify asymptomatic and symptomatic patients with pre-excitation on ECG using a prospective referral study of 178 patients, not restricted to athletes, between 2017 and 2021. Of the 164 who completed the exercise stress testing and EP studies, they found that exercise stress testing was not sufficient to rule out high-risk patients: six patients with apparently low-risk exercise findings nevertheless had an accessory-pathway refractory period or shortest pre-excited RR interval of 250 ms or less during EP testing, illustrating that abrupt loss of pre-excitation during exercise does not invariably exclude a high-risk pathway. Notably, they also reported a similar rate of inducible arrhythmias between both asymptomatic and symptomatic groups (61% vs. 69%, respectively), and that 41% of them had high-risk features on EP study after infusion of isoproterenol. They subsequently argued that all patients with ventricular pre-excitation should be considered for EP study with routine isoproterenol evaluation [67].

Evaluation should include a careful history, physical examination, echocardiography, and specialist discussion of accessory-pathway risk. Current guidance permits asymptomatic athletes with WPW pattern to continue participation during evaluation, while sports cardiology and electrophysiology specialists discuss noninvasive versus invasive assessment and, when indicated, ablation. This permission does not remove the need to complete pathway risk assessment, and athletes with WPW syndrome require electrophysiology evaluation [10,37].

## 14. Long QT Syndrome

Long QT syndrome is a heterogeneous group of channelopathies associated with prolonged QT on ECG (QTc > 460 ms in prepuberty, >470 ms male, >480 ms female) and is the most common cause of inheritable arrhythmias. However, despite that, it remains an uncommon cause of SCA/SCD in athletes [68,69]. In a longitudinal study of 494 athletes with LQTS between 2000 and 2020, only 2 died during follow-up, and both deaths were not attributed to LQTS [69]. Current guidelines support a return to play with close follow-up and beta-blocker therapy [10,37]. New gene therapies are currently under development, and more work will be needed to determine if these will impact risk [70].

## 15. Catecholaminergic Polymorphic Ventricular Tachycardia

Catecholaminergic polymorphic ventricular tachycardia is a rare inherited syndrome classically characterized by bidirectional/polymorphic VT in the setting of catecholamine surges, such as exercise or stress. It may be underreported in the literature and autopsy studies, as it is associated with structurally normal hearts and normal baseline ECGs [71]. Some estimates attribute up to 15% of AN-SUD in young individuals to CPVT [71,72]. Recently, Cho et al. ([71]) performed a retrospective analysis on 39 symptomatic pediatric patients with CPVT and found that 28 (71.8%) had an initial presenting symptom of syncope (20 of which were cardiogenic) and 11 (28.2%) had an aborted cardiac arrest [71]. Current guidelines recommend maximum effort exercise stress testing that includes burst efforts in the protocol [10,37,73]. In those with exercise-induced ventricular arrhythmias, beta-blockers and flecainide should be initiated, and consideration of left cardiac sympathetic denervation (LCSD) should be made for those who are at high risk or desire to participate in sports [37]. In Cho et al.’s observational study, triple treatment of beta-blocker, flecainide, and LCSD was effective in reducing breakthrough cardiac events but did not show a statistically significant impact on the incidence of SCD [71].

Current guidelines report that return to play can be considered in asymptomatic athletes, or once breakthrough events have resolved and the exercise test has normalized on optimized therapy, as risk is lowered. In athletes who have ongoing symptomatic CPVT (or complex ectopy) despite treatment (double or triple therapy), return to play is potentially harmful and should be avoided [2,37,74,75].

## 16. Myocarditis

Myocarditis remains a clinically relevant etiology of SCA and SCD in athletes with recent estimates suggesting 6–7% of SCA/SCD in young athletes [10,18]. Diagnosis relies on clinical symptoms such as chest pain, arrhythmias, and heart failure as well as diagnostic testing which includes ECGs, laboratory tests, echocardiography, and CMR. Myocarditis has a spectrum of different etiologies, including infectious (often viral), autoimmune, drugs, radiation, and genetics. Some athletes may be at higher risk of myocarditis due to several unique factors including sleep deprivation, jet lag, extreme temperature exposures, inflammation, medications, performance-enhancing substances, and immune suppression [76]. Since exercise may worsen myocardial inflammation, strenuous activity is restricted at the time of diagnosis during active myocarditis. There have been several recent key documents addressing return-to-play strategies that vary in their formal recommendations.

The 2024 ACC Expert Consensus Decision Pathway on Myocarditis generally advises abstaining from strenuous activity and competitive sport for 3–6 months in symptomatic (stage C/D) myocarditis, at which time the athlete will need to be reassessed for symptoms, arrhythmias with cardiac monitor, and evidence of myocardial inflammation or dysfunction with CMR. If the athlete has no symptoms, no arrhythmias, and CMR shows normal myocardial function, then a maximum-effort treadmill test is recommended prior to return to participation. If initial reassessment at 3–6 months is abnormal, then it is recommended that the athlete abstain from strenuous activity for another 3 months and undergo repeat evaluation [77].

The 2025 ESC Guidelines for the Management of Myocarditis and Pericarditis move away from the 3–6-month time period and describe at least a month without strenuous physical activity until clinical remission. Remission may occur at different time points and is assessed by clinical, laboratory, electrical, imaging, and functional findings. Laboratory markers include CRP and troponin levels. Electrical assessment includes ECG and ambulatory monitoring while imaging includes repeat CMR to identify presence of LGE and changes in T1, T2, and ECV values from initial study. The presence of LGE has been associated with patients with sudden cardiac death [78].

The 2025 AHA/ACC Clinical Considerations for Competitive Sports Participation for Athletes with Cardiovascular Abnormalities provides additional recommendations and guidance on the subject. For athletes with reduced LV function at the time of diagnosis, a period of 3 months without symptoms and with normalization of LV function, normalization of biomarkers, and lack of myocardial inflammation on CMR is recommended. For athletes with normal LV function at the time of diagnosis, resumption of strenuous activity is reasonable as early as 4–6 weeks if there are no signs of inflammation as above. For athletes with persistent LGE but resolution of edema by T2 signal on CMR, this pattern is consistent with prior myocarditis and participation in sports may be reasonable if asymptomatic with normal LV function [37,76].

## 17. Screening Guidelines, ECG Patterns, and Targeted Imaging

Screening with history and physical is universally recommended; however, ECG-specific screening is still disputed among different sports and legislation groups due to the high variability of results [2,4,37]. There have been multiple revisions to the ECG criteria over the past several decades with improvement in false-positive rates from the 2010 ESC criteria, through the 2013 Seattle criteria and 2014 Revised criteria to our most recent publication, the 2017 International Criteria [79]. The false-positive rates are dependent on the athlete population they are being applied to (adolescent, collegiate, masters) as well as race [80,81,82]. When comparing the AHA 14-point history and physical model to ECG screenings, a recent prospective study showed that AHA-14 only identified abnormalities in 43.8% of athletes with an SCA/SCD-associated condition, while ECG detected 93.8% [83]. To further investigate the diagnostic yield of ECG screening, a recent study screened 104,369 young individuals (62% male, 89% white, 9% athletes) from 2008 to 2018 with a questionnaire, ECG, and selective echocardiogram. Of these, 5.5% of screening tests yielded an abnormal result, with 2.5% referred for further evaluation revealing 280 conditions associated with SCA/SCD. Overall, ECG screening in this primarily non-athlete population was 75.4% sensitive for identifying conditions associated with SCA/SCD, while the questionnaire was only 32.5% sensitive [84]. It is worth distinguishing that the detection of the disease does not always equate to the prevention of SCD. There are also consequences of false-positive ECG tests, which often lead to additional cardiovascular testing, anxiety, cost, and temporary restriction from athletics until evaluation is complete.

The 2017 International Criteria describe abnormal ECG findings as T-wave inversion ≥ 1 mm in two or more contiguous lateral, inferolateral, or inferior leads (or in V2–V4 in athletes aged ≥16 years, other than the anterior variant described below), ST-segment depression, pathologic Q waves, prolonged QTc (≥470 ms in men or ≥480 ms in women), type 1 Brugada pattern, complete left bundle branch block (LBBB), nonspecific intraventricular conduction delay with QRS duration ≥ 140 ms, ventricular pre-excitation, epsilon wave, and two or more premature ventricular contractions on a resting 10 s tracing, among others. A finding of low-voltage QRS can be a red flag sign that can indicate inflammation, scarring, myocardial disease, or pericardial effusion [28,85,86]. These findings require further evaluation, which may include cardiac imaging and exercise testing though they do not all carry the same prognostic weight as it pertains to risk of SCD. Normal ECG findings include isolated QRS voltage criteria for LVH or RVH (voltage accompanied by T-wave inversion, ST-segment depression, or pathologic Q waves is not considered normal), incomplete right bundle branch block, early repolarization, anterior T-wave inversion confined to V1–V3 in athletes younger than 16 years, sinus bradycardia ≥ 30 bpm, first-degree AV block, and ectopic atrial or junctional rhythm [28,87,88]. There has been additional research on the ECG pattern historically referred to as the “black athlete” pattern characterized by J-point elevation and convex (“domed”) ST-segment elevation followed by T-wave inversion in V1–V4. This has also been described in non-black athletes, though at a lower prevalence. In a recent retrospective study of athletes with this pattern, there were no identified cardiac pathologies or breakthrough cardiovascular events. As such, it has been proposed to call this ECG pattern “athletic anterior early repolarization” [88]. Certain pathologies, such as coronary anomalies, aortic aneurysms, and some channelopathies, can present without overt ECG changes.

Imaging with echocardiogram can add to the diagnostic yield in cardiac screening of athletes. Oxborough et al. ([89]) cite estimates that up to 16% of athlete SCD cases involve structural disease without accompanying ECG abnormalities [89]. Echocardiogram can identify these pathologies if patient history discloses more risk than what is seen with initial ECG. In adolescents, follow-up imaging can add 1.2–4.5% yield with consideration for CMR for characterization of suspected myocardial tissue abnormalities [89]. As with ECGs, the echocardiogram can improve detection of pathologies but may not ultimately predict or prevent SCD in athletes. It also comes at additional cost and imaging should be interpreted by a cardiologist familiar with exercise-induced cardiac remodeling so as to reduce unnecessary additional testing and restriction from participation.

For athletes with identified pathologies, several exercise testing modalities can be utilized to evaluate capacity to perform strenuous activity and potential for SCD. Stress echocardiogram can be used to assess for stress-induced regional wall motion abnormalities in anomalous coronary arteries or severity of LVOT obstruction in athletes with HCM. CPET can be used to assess cardiorespiratory function in individuals with congenital heart disease. Treadmill ECG is useful for the assessment of underlying arrhythmogenic potential of certain substrates.

## 18. Emergency Preparedness and Immediate Management of Sudden Cardiac Arrest

Despite improved cardiovascular screening methods and risk stratification, the potential for sudden cardiac arrest cannot be entirely eliminated. The early recognition of medical emergencies and prompt response is critical to athlete survival [90]. It is imperative that schools, organizations, venues, etc., have established emergency action plans that are coordinated with local emergency services [91].

Emergency action plans (EAPs) should be established for each venue in which athletes practice and compete. There should be careful consideration for environmental factors as well as recognition of logistical or physical barriers that may delay access to emergency services. EAPs should be practiced annually and involve coaches, trainers, athletes, administrative personnel and any other individual who may be present [91].

When evaluating a collapsed athlete, the presumptive cause of the collapse should be cardiac. It is important to recognize that collapsed athletes may have abnormal breathing and movements which can be present in sudden cardiac arrest and are not necessarily indicative of a pulmonary or neurologic cause of collapse. Pulmonary embolism belongs in the differential diagnosis of sudden collapse and all-cause sudden death in athletes, but considering it must not delay CPR or AED use. Assuming the event is cardiac avoids delays in obtaining AED and improves chances of survival [90,91].

The recommended interval from athlete collapse to AED use is less than 3 min [92]. AEDs should be appropriately positioned to achieve this mark. Bystander presence, bystander CPR, and bystander AED use are all associated with athlete survival [93]. Multiple studies have demonstrated that bystander AED is the strongest independent predictor of survival. Survival rates approach 85–90% with bystander CPR and AED use [94,95]. Over the years, there has not been a significant change in reported SCA events; however, some communities have seen an increase in bystander CPR from ~35% to ~95%, increase in public AED use from ~2% to ~29%, and survival at hospital discharge from ~24% to ~67% [96].

The application of the AED should be rehearsed, especially for those athletes who wear protective gear such as football or lacrosse. The shoulder pads can generally be opened/split rapidly to expose undergarments which will also need to be cut or removed to properly place the AED pads on bare skin. As part of the EAP, one member should promptly start CPR while another member works to remove equipment and a third member retrieves the AED and applies it promptly [90,91].

Post cardiac arrest care continues at the appropriate medical facility where the athlete should undergo comprehensive cardiovascular evaluation to determine the etiology of sudden cardiac arrest with expert consultation with electrophysiology to discuss the role of ICD for secondary prevention based on findings.

## 19. Survival Evaluation, ICD Indications and New Devices

After stabilization, a survivor needs a structured etiologic assessment that includes a thorough review of arrest circumstances, witness accounts, available rhythm recordings, review of any available video recordings of the event, medication and substance exposure, personal and three-generation family history, and laboratory assessment for reversible causes. Serial ECGs and continuous rhythm monitoring should be reviewed for arrhythmias and clues to conditions like long QT, WPW, Brugada or acute ischemia. All survivors should be evaluated by echocardiography to assess ventricular function, cardiomyopathy, valvular or other structural abnormalities. CMR with LGE assessment is recommended when the cause remains unexplained, to identify occult myocardial disease or scar. CCTA is useful in appropriately selected patients to screen for coronary artery disease, dissection or anomalous coronary origins. Further evaluation with stress testing when clinically stable (treadmill exercise and/or specific pharmacologic testing with agents such as adenosine for suspicion of WPW and procainamide for Brugada) can be considered with careful monitoring. Phenotype-guided genetic testing, with genetic counseling, is recommended when an inherited disorder is suspected and results could influence diagnosis, management, or family screening; channelopathy and cardiomyopathy testing may also be considered in selected survivors whose arrest remains unexplained [2,10,37].

Current guidelines recommend that in athletes who have experienced SCA, an ICD should be considered based on underlying disease entity, and that it is reasonable to consider sport type and disease entity when considering ICD type and location. While most athletes with SCA are treated with an ICD, notable exceptions can include those with reversible conditions such as ablated WPW syndromes, commotio cordis, or surgically corrected coronary anomalies [2]. Those with CPVT require an experienced cardiac electrophysiologist as ICD shocks can be pro-rhythmic in these patients [2,37].

Return to play following ICD implantation may be reasonable through shared decision-making and consideration of disease etiology [2,37]. A recent retrospective review of publicly reported cases identified 37 elite athletes with ICDs, most implanted for secondary prevention, of whom 25 returned to sport; clinical outcomes, procedural risk, recurrence, and psychological impact varied, and this selected series is subject to reporting bias and cannot establish a return-to-sport rate [97]. For ICDs with transvenous leads, athletes must often wait at least 4–6 weeks to allow transvenous-lead healing before consideration of returning to play [2]. Contemporary data support subcutaneous ICD (S-ICD) as a reasonable option in appropriately selected patients who do not require bradycardia pacing, cardiac resynchronization, or antitachycardia pacing (ATP) [98,99].

Emerging extravascular and modular leadless pacing–defibrillator systems may be especially attractive for young athletes by combining avoidance of transvenous leads with anti-tachycardia pacing; however, athlete-specific experience, collision-sport safety and long-term lead or device durability remain limited [100,101]. Wearable cardioverter-defibrillators may provide temporary protection in selected patients with elevated arrhythmic risk while recovery or the need for permanent ICD implantation is assessed, and newer patch-based systems have also undergone prospective clinical evaluation [102,103].

## 20. Artificial Intelligence in Risk Prediction and Screening

Artificial intelligence (AI) with deep learning, including convolutional neural networks applied to ECG data and large language models applied to text, has been explored in a variety of cardiovascular applications to SCA/SCD in the general public. These applications include disease detection, event prediction, and clinical information synthesis, which require different forms of validation. Large language models may also support documentation and information synthesis under clinician oversight. However, these capabilities have largely not been validated for interpretation of physiological signals or prediction of SCA/SCD in athletes [104,105].

As an example of AI use in SCA/SCD in the general public, Obermeyer et al. ([106]) recently applied a deep learning model to a dataset linking all ECGs in a Swedish region to death certificates, with external testing in a US health system and a Taiwanese hospital case–control dataset. They found that high-risk morphologies of QRS patterns often exhibit axis deviation or a rotation in the average electrical vector of depolarization, such as left axis deviation (LAD) or left anterior fascicular block (LAFB) in the frontal plane and posterior rotation or “poor R wave progression” in the axial plane. Their model also identified a unique high-risk morphology of aVL, where the terminal aspect of the R wave is slurred. These findings have not yet been studied specifically in athletes [106].

Athlete-specific studies illustrate the limitations of transferring AI models between populations. Palermi et al. ([104]) externally evaluated hospital-trained AI-ECG models in 1115 competitive athletes undergoing pre-participation screening. The target was imaging-confirmed structural heart disease, rather than SCA/SCD. Discrimination declined to an AUROC of 0.70 for valvular heart disease and 0.69 for cardiomyopathies. Positive predictive values were ≤8% at the evaluated thresholds despite negative predictive values of approximately 99% [104]. In a separate retrospective multicenter study of 2950 competitive athletes, a general-purpose ChatGPT (OpenAI, San Francisco, CA, USA) model (Version 5) interpreting ECG images without task-specific training achieved an AUC of 0.52 for confirmed cardiovascular disease and missed approximately 79% of affected athletes at the selected threshold [107]. These studies suggest that AI models developed in general clinical populations may not transfer to athletes, and neither evaluated SCA/SCD prevention nor sports-clearance outcomes [105,107].

Outside of ECGs, AI and computational modeling have been explored using cardiac imaging, genetic information and clinical data in the general population. Shiri et al. ([40]) developed fully automated AI for the detection and classification of anomalous coronary arteries using 3D-CCTA images from clinical cohorts. Their models underwent internal testing, including a prospective cohort, and had an AUC of 0.99 or more for detecting AAOCA with similarly excellent sensitivities and specificities (0.95–0.99) [40]. These results have not yet been validated in athletes.

A promising new avenue in the intersection of AI and cardiology that could eventually inform SCA/SCD assessment is the development of cardiac digital twins (CDT). One of the first proof-of-concept uses of a cardiac digital twin was a study by Arevalo et al. ([108]), where they created a personalized 3D model of the heart using CMR and computational modeling to assess the risk of significant arrhythmia, appropriate ICD therapy or cardiac death [108]. With the advancement of AI, the development of cardiac digital twins is easier than before, and they are often created with a proprietary combination of imaging and clinical information with AI to create a 3D model of the heart [109]. The use of CDTs has not yet been studied in the athlete population.

Despite potential as a simple and easily performed screening tool, current data on outcomes of various AI models remain limited. Various models with different clinical criteria and incorporation of different patient media have various results. Several prospective studies are small and short-term, making generalizability questionable [104]. External validation and model calibration vary in reporting and reproducibility. Before use in athlete screening, models require independent validation in representative athletic populations, assessment of calibration and predictive values at the intended disease prevalence, and evaluation of downstream testing with outcomes. Prospective studies are needed before these tools can be relied upon to guide sports clearance [105].

## 21. Conclusions

Sudden cardiac arrest and death in athletes is an uncommon yet devastating consequence of a spectrum of structural, electrical, congenital, and acquired disease. Contemporary research has further clarified known risk factors, such as coronary anomalies and genetic disorders, while illuminating recently discovered risk factors like myocardial fibrosis. Screening and disease-specific evaluation can identify actionable conditions, but they do not predict every arrest. Every training and competition setting therefore needs practiced emergency procedures, immediate CPR, and rapid AED access. Current post-arrest assessments rely on multiple modalities and use the information from them to create an individualized evaluation with longitudinal surveillance and shared decision-making regarding return to play. ICDs remain a cornerstone in secondary prevention in appropriate conditions. Ultimately, the goal is to make participation in sports as safe as possible through individualized care, informed shared decision-making, and a practiced, timely response to cardiac arrest.

## Data Availability

No new data were created or analyzed in this study. Data sharing is not applicable to this article.

## Abbreviations

The following abbreviations are used in this manuscript:

AAOCA	anomalous aortic origin of a coronary artery
AAOLCA	anomalous aortic origin of the left coronary artery
AAORCA	anomalous aortic origin of the right coronary artery
ACC	American College of Cardiology
ACM	arrhythmogenic cardiomyopathy
AHA	American Heart Association
AI	artificial intelligence
AN-SUD	autopsy-negative sudden unexplained death
ASD	atrial septal defect
CAC	coronary artery calcium
CAD	coronary artery disease
CCTA	coronary computed tomography angiography
CDT	cardiac digital twin
CMR	cardiac magnetic resonance imaging
CPET	cardiopulmonary exercise testing
CPVT	catecholaminergic polymorphic ventricular tachycardia
DCM	dilated cardiomyopathy
DSP	desmoplakin
ECG	electrocardiogram
EF	ejection fraction
EP	electrophysiology
ESC	European Society of Cardiology
HCM	hypertrophic cardiomyopathy
ICD	implantable cardioverter-defibrillator
IVF	idiopathic ventricular fibrillation
LAD	left axis deviation
LAFB	left anterior fascicular block
LBBB	left bundle branch block
LCSD	left cardiac sympathetic denervation
LGE	late gadolinium enhancement
LQTS	long QT syndrome
LV	left ventricle
LVEF	left ventricular ejection fraction
LVH	left ventricular hypertrophy
LVOT	left ventricular outflow tract
MET	metabolic equivalent of task
MI	myocardial infarction
NCAA	National Collegiate Athletic Association
NSVT	nonsustained ventricular tachycardia
PAH	pulmonary arterial hypertension
PKP2	plakophilin-2
POST SCD	Postmortem Systemic Investigation of Sudden Cardiac Death
PVC	premature ventricular contraction
RBBB	right bundle branch block
RV	right ventricle
SCA	sudden cardiac arrest
SCD	sudden cardiac death
S-ICD	subcutaneous implantable cardioverter-defibrillator
TTE	transthoracic echocardiography
VF	ventricular fibrillation
VSD	ventricular septal defect
VT	ventricular tachycardia
WPW	Wolff-Parkinson-White
